# The most influential papers in unicompartmental knee arthroplasty

**DOI:** 10.1186/s43019-020-00072-1

**Published:** 2020-10-09

**Authors:** Lukas A. Holzer, Gerold Holzer

**Affiliations:** 1 Department of Orthopedic Surgery, AUVA Trauma Center Klagenfurt, Waidmannsdorferstraße 35, 9020 Klagenfurt am Wörthersee, Austria; 2 Department of Orthopedics and Traumatology, Medical University of Vienna, Vienna, Austria

**Keywords:** Unicompartmental knee arthroplasty, Unicondylar knee arthroplasty, Bibliometric study, Citation analysis

## Abstract

**Purpose:**

Unicompartmental knee arthroplasty (UKA) is a treatment option for anteromedial osteoarthritis of the knee. The number of UKA has been increasing constantly worldwide in recent decades. The aim of this study was to determine the most frequently cited scientific articles addressing this subject and to establish a ranking of the 50 most influential papers.

**Methods:**

The 50 most cited articles related to UKA were searched in Web of Science® (Clarivate Analytics, Penn., USA) by the use of defined search terms. All types of scientific papers with reference to this topic were ranked according to the absolute number of citations and analyzed for the following characteristics: journal title, year of publication, number of citations, citation density, geographic origin, article type, and level of evidence.

**Results:**

The 50 most cited articles had up to 453 citations. Most papers were published in the *Journal of Bone and Joint Surgery* (British volume). More than half of the articles were published in the 2000s and 2010s (*n* = 30). Ten countries contributed to the top 50 list, with most contributions from the UK (*n* = 17). Most articles could be attributed to the category of Clinical Science (*n* = 33), and most reported level IV studies.

**Conclusion:**

Most of the frequently cited articles in UKA are clinical studies that have a low level of evidence. Few basic scientific studies could be identified, which suggests that most product development is done by commercial companies.

## Introduction

Unicompartmental knee arthroplasty (UKA) dates back to the 1950s, following the introduction of the MacIntosh and McKeever hemiarthroplasty [1]. Since then, patient management, implant design, and surgical techniques, such as patient-specific instrumentation, have advanced [1, 2]. Nowadays, UKA is considered a less-invasive treatment option for anteromedial osteoarthritis than total knee (TKA) arthroplasty [3–5]. As a result, the number of UKA has been increasing constantly worldwide in recent decades [6–8]. Due to the clinical relevance of UKA, numerous scientific papers related to this topic were published, that is, more than 2000 articles.

A citation is a reference or a quotation from previous scientific work that has been published in books or scientific journals [9]. The number of citations of published scientific articles is considered a parameter of its influence and impact in the scientific community. The impact factor of a journal is a widely accepted as a parameter of scientific quality and importance. It is calculated by the number of citations [9].

Analyses of citations in a specific scientific field allow us to give an overview of the most influential articles and offer physicians, researchers, and residents insight on the relevant current literature. Such studies have been done in different medical disciplines such as orthopedic surgery or general surgery; in a variety of orthopedic subspecialties including arthroplasty, arthroscopy, or hand surgery; and for conditions such as osteoporosis or anterior cruciate ligament rupture [10–21].

The aim of this study was to determine the most cited scientific articles related to UKA and to establish a ranking of the most influential papers by the use of the Web of Science® database.

## Material and methods

### Search strategy

In March 2020, Web of Science® (Clarivate Analytics, Penn., USA) was searched for the following search terms “Unicompartmental Knee Arthroplasty,” “Unicondylar Knee Arthroplasty,” “Unicompartmental Knee Replacement,” “Knee Arthroplasty,” “Knee Replacement,” “Implant,” and “Prosthesis.”

The search output was documented after completion of the search. All scientific articles related to UKA that could be identified were included and ranked according to the absolute number of citations (times cited in the Web of Science core collection). If the absolute number of citations was the same in two or more papers, the publication that had the higher citation density (see below) was ranked higher. The fifty most cited articles were chosen and represent the list of the most influential papers in UKA. The literature search and subsequent data analysis were done by a board-certified orthopedic surgeon.

### Data analysis

The fifty most cited articles were studied in detail for the following characteristics: article title, journal title, year of publication, and origin of the corresponding author. Each publication was assigned to a single country of origin. This decision was based on the corresponding author’s address, as the corresponding author is in charge of the article and the guarantor of the data [22].

A citation density (the number of citations per year since publication) was calculated to show the relative impact of the included articles [17].

The included articles were allocated to different scientific categories as follows: “Clinical Science,” “Basic Science,” “Registry & Database,” and “Review.”

The level of evidence was assessed in all articles that were attributed to the Clinical Science category. This assessment was done according to the guidelines for clinical articles by the Oxford Centre for Evidence-Based Medicine 2016 Levels of Evidence [23].

## Results

The absolute number of citations for the fifty most cited articles on UKA range from 110 to 453 times. The fifty articles were cited, in total, 8436 times. The mean number of citations of an included paper was 169 times (± 69). The top ten papers reached at least 219 citations since publication.

The absolute numbers of citations for the fifty most influential articles on UKA are shown in Table 1, and the top ten papers, according to their relative number of citations (highest citation density), are presented in Table 2.

**Table 1:** The fifty highest cited papers in unicompartmental knee arthroplasty

Rank	Article	Absolute number of citations	Level of evidence
1	Murray DW, Goodfellow JW, O’Connor JJ. The Oxford medial unicompartmental arthroplasty - a ten-year survival study. J Bone Joint Surg Br. 1998;80B:983–989.	453	IV
2	Robertsson O, Dunbar M, Pehrsson T, Knutson K, Lidgren L. Patient satisfaction after knee arthroplasty - a report on 27,372 knees operated on between 1981 and 1995 in Sweden. Acta Orthop Scand. 2000;71:262–267.	346	
3	Kozinn SC, Scott R. Current concepts review - unicondylar knee arthroplasty. J Bone Joint Surg Am. 1989;71A:145–150.	305	
4	Engh GA, Dwyer KA, Hanes CK. Polyethylene wear of metal-backed tibial components in total and unicompartmental knee prostheses. J Bone Joint Surg Br. 1992;74:9–17.	284	
5	Svard UCG, Price AJ. Oxford medial unicompartmental knee arthroplasty - A survival analysis of an independent series. J Bone Joint Surg Br. 2001;83B:191–194.	258	IV
6	Berger RA, Meneghini RM, Jacobs JJ, Sheinkop MB, Della Valle CJ. Results of unicompartmental knee arthroplasty at a minimum of ten years of follow-up. J Bone Joint Surg Br. 2005;87A:999–1006.	247	IV
7	Labek G, Thaler M, Janda W, Agreiter M, Stockl B. Revision rates after total joint replacement cumulative results from worldwide joint register datasets. J Bone Joint Surg Br. 2011;93B:293–297.	244	
8	Jamsen E, Huhtala H, Puolakka T, Moilanen T. Risk factors for infection after knee arthroplasty a register-based analysis of 43,149 cases. J Bone Joint Surg Am. 2009;91A:38–47.	233	
9	Price AJ, Webb J, Topf H, Dodd CAF, Goodfellow JW, Murray DW. Rapid recovery after oxford unicompartmental arthroplasty through a short incision. J Arthroplasty. 2001;16:970–976.	232	III
10	Newman JH, Ackroyd CE, Shah NA. Unicompartmental or total knee replacement? Arthritis five-year results of a prospective, randomised trial of 102 osteoarthritic knees with unicompartmental arthritis. J Bone Joint Surg Br. 1998;80B:862–865.	219	I
11	Knutson K, Lewold S, Robertsson O, Lidgren L. The Swedish knee arthroplasty register - a nationwide study of 30,003 knees 1976–1992. Acta Orthop Scand. 1994;65:375–386.	197	
12	Argenson JNA, Chevrol-Benkeddache Y, Aubaniac JM. Modern unicompartmental knee arthroplasty with cement - a three to ten-year follow-up study. J Bone Joint Surg Am. 2002;84A:2235–2239.	187	IV
13	Hernigou P, Deschamps G. Alignment influences wear in the knee after medial unicompartmental arthroplasty. Clin Orthop Relat Res. 2004;423:161–165.	180	IV
14	Cartier P, Sanouiller JL, Grelsamer RP. Unicompartmental knee arthroplasty surgery - 10-year minimum follow-up period. J Arthroplasty. 1996;11:782–788.	179	IV
15	Goodfellow JW, Kershaw CJ, Benson MKD, O’Connor JJ. The Oxford knee for unicompartmental osteoarthritis. The first 103 cases. J Bone Joint Surg Br. 1988;70:692–701.	175	IV
16	Newman J, Pydisetty RV, Ackroyd C. Unicompartmental or total knee replacement the 15-year results of a prospective randomised controlled trial. J Bone Joint Surg Br. 2009;91B:52–57.	172	I
17	Laurencin CT, Zelicof SB, Scott RD, Ewald FC. Unicompartmental versus total knee arthroplasty in the same patient - a comparative-study. 1991;273:151–156.	169	III
18	Pandit H, Jenkins C, Gill HS, Barker K, Dodd CAF, Murray DW. Minimally invasive Oxford phase 3 unicompartmental knee replacement results of 1000 cases. J Bone Joint Surg Br. 2011;93B:198–204.	167	II
19	Price AJ, Svard U. A second decade lifetable survival analysis of the Oxford unicompartmental knee arthroplasty. 2011;469:174–179.	161	IV
20	Pandit H, Jenkins C, Barker K, Dodd CAF, Murray DW. The Oxford medial unicompartmental knee replacement using a minimally-invasive approach. J Bone Joint Surg Br. 2006;88B:54–60.	160	II
21	Liddle AD, Judge A, Pandit H, Murray DW. Adverse outcomes after total and unicompartmental knee replacement in 101,330 matched patients: a study of data from the National Joint Registry. Lancet. 2014;384:1437–1445.	158	
22	Brocklehurst R, Bayliss MT, Maroudas A, Coysh HL, Freeman MAR, Revell PA, Ali SY. The composition of normal and osteoarthritic articular cartilage from human knee joints. With special reference to unicompartmental replacement and osteotomy of the knee. J Bone Joint Surg Am. 1984;66A:95–106.	158	
23	Robertsson O, Knutson K, Lewold S, Lidgren L. The routine of surgical management reduces failure after unicompartmental knee arthroplasty. J Bone Joint Surg Br. 2001;83B:45–49.	157	
24	Scott RD, Santore RF. Unicondylar unicompartmental replacement for osteoarthritis of the knee. J Bone Joint Surg Am. 1981;63:536–544.	157	IV
25	Price AJ, Waite JC, Svard, U. Long-term clinical results of the medial Oxford unicompartmental knee arthroplasty. Clin Orthop Relat Res. 2005;435;171–180.	150	IV
26	Blunn GW, Joshi AB, Minns RJ, Lidgren L, Lilley P, Ryd L, Engelbrecht E, Walker PS. Wear in retrieved condylar knee arthroplasties. A comparison of wear in different designs of 280 retrieved condylar knee prostheses. J Arthroplasty. 1997;12:281–290.	148	
27	White SH, Ludkowski PF, Goodfellow JW. Anteromedial osteoarthritis of the knee. J Bone Joint Surg Br. 1991;73:582–586.	147	
28	Cobb J, Henckel J, Gomes P, Harris S, Jakopec M, Rodriguez F, Barrett A, Davies B. Hands-on robotic unicompartmental knee replacement: A prospective, randomised controlled study of the acrobot system. J Bone Joint Surg Br. 2006;88B:188–197.	146	I
29	Barrett WP, Scott RD. Revision of failed unicondylar unicompartmental knee arthroplasty. J Bone Joint Surg Am. 1987;69A:1328–1335.	144	IV
30	Hernigou P, Deschamps G. Posterior slope of the tibial implant and the outcome of unicompartmental knee arthroplasty. J Bone Joint Surg Am. 2004;86A:506–511.	142	IV
31	Scott RD, Cobb AG, McQueary FG, Thornhill TS. Unicompartmental knee arthroplasty - 8-year to 12-year follow-up evaluation with survivorship analysis. Clin Orthop Relat Res. 1991;271:96–100.	138	IV
32	Marmor L. Unicompartmental knee arthroplasty. 10-year to 13-year follow-up-study. Clin Orthop Relat Res. 1988;226:14–20.	135	IV
33	Psychoyios V, Crawford RW, O’Connor JJ, Murray DW. Wear of congruent meniscal bearings in unicompartmental knee arthroplasty - A retrieval study of 16 specimens. J Bone Joint Surg Br. 1998;80B:976–982.	130	
34	Robertsson O, Bizjajeva S, Fenstad AM, Furnes O, Lidgren L, Mehnert F, Odgaard A, Pedersen AB, Havelin LI. Knee arthroplasty in Denmark, Norway and Sweden. A pilot study from the Nordic Arthroplasty Register Association. Acta Orthop. 2010;81:82–89.	124	
35	Berger RA, Nedeff DD, Barden RM, Sheinkop MM, Jacobs JJ, Rosenberg AG. Unicompartmental knee arthroplasty. Clinical experience at 6-to 10-year followup. Clin Orthop Relat Res. 1999;367:50–60.	122	II
36	Pennington DW, Swienckowski JJ, Lutes WB, Drake GN. Unicompartmental knee arthroplasty in patients sixty years of age or younger. J Bone Joint Surg Am. 2003;85A:1968–1973.	120	IV
37	Willis-Owen CA, Konyves A, Martin DK. Factors affecting the incidence of infection in hip and knee replacement an analysis of 5277 cases. J Bone Joint Surg Br. 2010;92B:1128–1133.	117	II
38	Collier MB, Engh CA, McAuley JP, Engh GA. Factors associated with the loss of thickness of polyethylene tibial bearings after knee arthroplasty. 2007;89A:1306–1314.	117	
39	Furnes O, Espehaug B, Lie SA, Vollset SE, Engesaeter LB, Havelin LI. Failure mechanisms after unicompartmental and tricompartmental primary knee replacement with cement. J Bone Joint Surg Am. 2007;89A:519–525.	116	
40	Koskinen E, Paavolainen P, Eskelinen A, Pulkkinen P, Remes V. Unicondylar knee replacement for primary osteoarthritis: a prospective follow-up study of 1819 patients from the Finnish Arthroplasty Register. Acta Orthop. 2007;78:128–135.	116	
41	Emerson RH, Higgins LL. Unicompartmental knee arthroplasty with the Oxford prosthesis in patients with medial compartment arthritis. J Bone Joint Surg Am. 2008;90A:118–122.	115	IV
42	Price AJ, Dodd CAF, Svard UGC, Murray DW. Oxford medial unicompartmental knee arthroplasty in patients younger and older than 60 years of age. J Bone Joint Surg Br. 2005;87B:1488–1492.	115	III
43	Padgett DE, Stern SH, Insall JN. Revision total knee arthroplasty for failed unicompartmental replacement. 1991;73A:186–190.	115	IV
44	Lyons MC, MacDonald SJ, Somerville LE, Naudie DD, McCalden RW. Unicompartmental versus total knee arthroplasty database analysis: Is there a winner? Clin Orthop Relat Res. 2012;470:84–90.	113	II
45	Berger RA, Kusuma SK, Sanders SA, Thill ES, Sporer SM. The feasibility and perioperative complications of outpatient knee arthroplasty. Clin Orthop Relat Res. 2009;467:1443–1449.	113	IV
46	Lombardi AV, Berend KR, Walter CA, Aziz-Jacobo J, Cheney NA. Is recovery faster for mobile-bearing unicompartmental than total knee arthroplasty? Clin Orthop Relat Res. 2009;467:1450–1457.	112	III
47	Willis-Owen CA, Brust K, Alsop H, Miraldo M, Cobb JP. Unicondylar knee arthroplasty in the UK National Health Service: an analysis of candidacy, outcome and cost efficacy. Knee. 2009;16:473–478.	111	III
48	Lewold S, Robertsson O, Knutson K, Lidgren L. Revision of unicompartmental knee arthroplasty: outcome in 1135 cases from the Swedish Knee Arthroplasty study. Acta Orthop Scand. 1998;69:469–474.	111	II
49	Broughton NS, Newman JH, Baily RAJ. Unicompartmental replacement and high tibial osteotomy for osteoarthritis of the knee - a comparative-study after 5–10 years. J Bone Joint Surg Br. 1986;68:447–452.	111	III
50	Riddle DL, Jiranek WA, McGlynn FJ. Yearly incidence of unicompartmental knee arthroplasty in the United States. J Arthroplasty. 2008;23:408–412.	110	IV

**Table 2:** The ten highest cited papers in unicompartmental knee arthroplasty in relative numbers

Rank	Article	Citation density	Level of evidence
1	Labek G, Thaler M, Janda W, Agreiter M, Stockl B. Revision rates after total joint replacement cumulative results from worldwide joint register datasets. J Bone Joint Surg Br. 2011;93B:293–297.	27,11	
2	Liddle AD, Judge A, Pandit H, Murray DW. Adverse outcomes after total and unicompartmental knee replacement in 101,330 matched patients: a study of data from the National Joint Registry. Lancet. 2014;384:1437–1445.	26,33	
3	Jamsen E, Huhtala H, Puolakka T, Moilanen T. Risk factors for infection after knee arthroplasty a register-based analysis of 43,149 cases. J Bone Joint Surg Am. 2009;91A:38–47.	21,18	
4	Murray DW, Goodfellow JW, O’Connor JJ. The Oxford medial unicompartmental arthroplasty - a ten-year survival study. J Bone Joint Surg Br. 1998;80B:983–989.	20,59	IV
5	Pandit H, Jenkins C, Gill HS, Barker K, Dodd CAF, Murray DW. Minimally invasive Oxford phase 3 unicompartmental knee replacement results of 1000 cases. J Bone Joint Surg Br. 2011;93B:198–204.	18,56	II
6	Price AJ, Svard U. A second decade lifetable survival analysis of the Oxford unicompartmental knee arthroplasty. 2011;469:174–179.	17,89	IV
7	Robertsson O, Dunbar M, Pehrsson T, Knutson K, Lidgren L. Patient satisfaction after knee arthroplasty - a report on 27,372 knees operated on between 1981 and 1995 in Sweden. Acta Orthop Scand. 2000;71:262–267.	17,3	
8	Berger RA, Meneghini RM, Jacobs JJ, Sheinkop MB, Della Valle CJ. Results of unicompartmental knee arthroplasty at a minimum of ten years of follow-up. J Bone Joint Surg Br. 2005;87A:999–1006.	16,47	IV
9	Newman J, Pydisetty RV, Ackroyd C. Unicompartmental or total knee replacement the 15-year results of a prospective randomised controlled trial. J Bone Joint Surg Br. 2009;91B:52–57.	15,63	I
10	Lyons MC, MacDonald SJ, Somerville LE, Naudie DD, McCalden RW. Unicompartmental versus total knee arthroplasty database analysis: Is there a winner? Clin Orthop Relat Res. 2012;470:84–90.	14,13	II

**Fig. 1:**
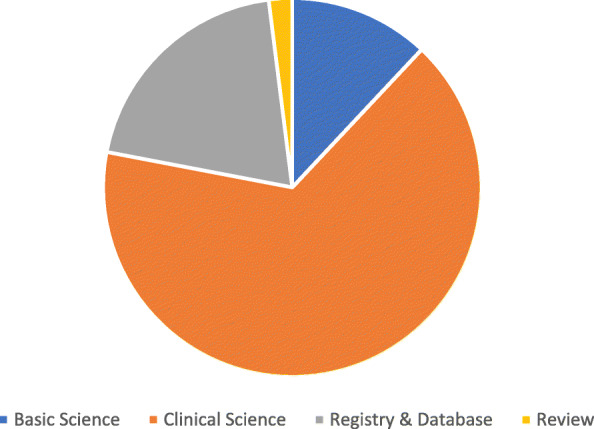
Distribution of categories

**Fig. 2:**
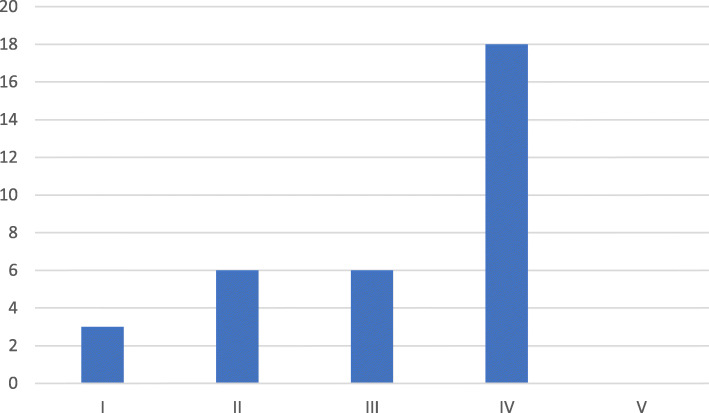
Distribution of the level of evidence in the clinical studies

Thirty-three articles were allocated to the “Clinical Science” category, which was the most frequently noted category. Figure 1 presents the distribution according to the different categories.Within the category “Clinical Science,” the level of evidence was analyzed. Eighteen studies of this category were categorized as level IV studies. The distribution of the level of evidence is shown in Fig. 2.

**Fig. 3:**
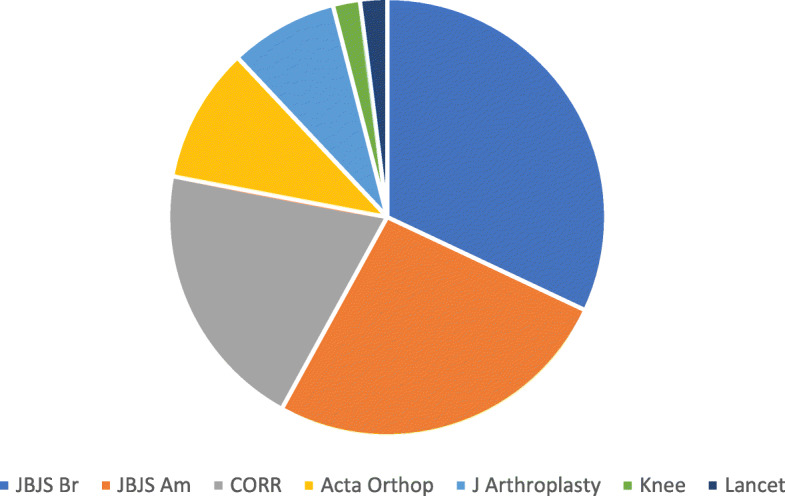
Distribution of the journals

The *Journal of Bone and Joint Surgery* (British volume) has published most papers of the list (*n* = 16). The *Journal of Bone and Joint Surgery* (American volume), *Clinical Orthopaedics and Related Research, Acta Orthopaedica (Scandinavica), Journal of Arthroplasty, Lancet,* and *Knee* were other journals that contributed to the list (Fig. 3).

Research institutions from the UK had the highest number of contributions (*n* = 17). Nine further countries contributed to the top 50 list: the USA with 16; Sweden with six; France with four; Finland with two; and Australia, Austria, Canada, India, and Norway with one each. Thirty-one publications were from Europe and 17 from North America.

**Fig. 4:**
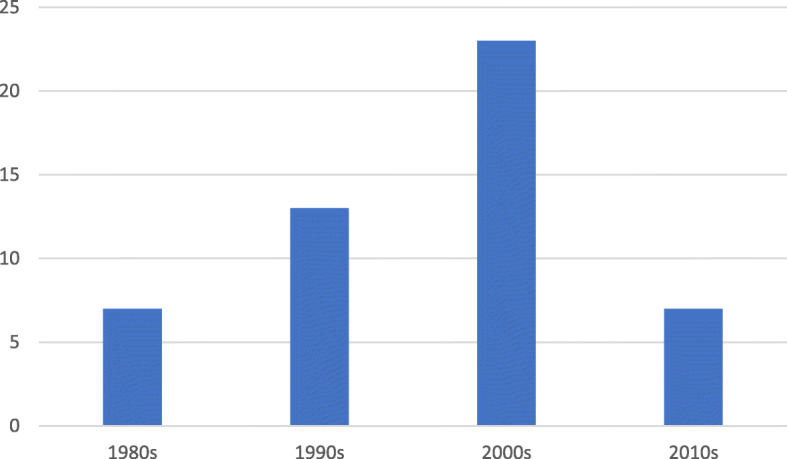
Distribution of articles according to publication decades

The publication years of these articles span 1981 to 2014. Figure 4 shows the decade in which the research was published. Most articles were published in the 2000s (*n* = 23), followed by the 1990s (*n* = 13), and then the 1980s and 2010s with seven publications per decade.

## Discussion

UKA is considered an alternative treatment option for anteromedial osteoarthritis to TKA. Because of the growing scientific interest in the field of UKA, we tried to identify the most influential articles related to UKA as guidance for clinical practice and future research. Publications on UKA were cited up to 453 times since publication, with the top ten articles in this field being cited at least 219 times. The citation number of articles in UKA is lower than in other fields of orthopedics, such as total hip and knee arthroplasty, cartilage surgery, and anterior cruciate ligament injury [13, 16, 20, 21]. However, compared to articles on hand surgery or pediatric orthopedics, articles on UKA are well cited [15, 18]. This indicates that the field of UKA is a highly driven industry such as the field of arthroplasty on the whole [24, 25].More than two-thirds of the articles were attributed to the category “Clinical Science.” Most of these were level IV studies, which indicates a low level of evidence in the field of UKA. This points to a need for clinical studies in UKA with better study design so as to gain more evidence in the future. Only three articles were level I studies. Two of these were prospective randomized controlled trials comparing UKA and TKA in the treatment of osteoarthritis of the knee.

The second most frequent category was “Registry and Database,” with ten articles. This indicates the great interest in implant performance and survival. Only six papers were in the category “Basic Science.” Product developments in the field of UKA are mostly done by commercial companies. These products are then used in clinical practice and studied clinically. Interestingly, the most highly cited contributions in the field of UKA were from the UK (*n* = 17). This is in contrast to most bibliometric analyses done so far, in which the USA contributed most of the scientific work [10–21]. Furthermore, in the field of UKA, a high number of contributions are from European countries, especially Scandinavian ones. This is also confirmed by the publishing journals. A large proportion of the articles was published in the *Journal of Bone and Joint Surgery* (British volume). Another seven articles were published in European journals. This finding is also in contrast to other bibliometric analyses in orthopedic surgery so far [10–21]. Interestingly, most articles have been published since 2000. This indicates an increasing trend in UKA in the last recent decades.

In general, bibliometrics has its limitations. The identification of the fifty most cited papers still remains a selection, even when using predefined search criteria. In this study, the absolute number of citations was considered an objective parameter. Among the scientific community, the impact factor is an accepted parameter of an article’s influence. The impact factor is directly influenced by the number of citations. However, the citation numbers of articles can be manipulated by various factors such as self-citations (in small numbers) and therefore might not necessarily represent the objective value of scientific work [9].

The literature search was performed in the Web of Science®. Textbooks, doctoral theses, presentations, and new media are not included in this database. Therefore, citations of articles of such sources might have been missed.

Another limiting factor might be the cross-sectional study design based on total citation numbers. As a result, more recent influential papers might not have been identified and were not considered eligible for the list.

## Conclusions

This study provides a list of the fifty most influential articles on UKA, which will help physicians and scientists to obtain an overview on past and current trends in the field of UKA. Most articles could be attributed to the category Clinical Science and provided a low level of evidence. This provides a basis for both further discussion and highlights the need for future research.

## Data Availability

The datasets developed during and/or analyzed during the current study are available from the corresponding author on reasonable request.
